# Antimicrobial susceptibility testing and tentative epidemiological cut-off values for *Lactobacillaceae* family species intended for ingestion

**DOI:** 10.3389/frabi.2023.1162636

**Published:** 2023-06-15

**Authors:** Katrine Nøhr-Meldgaard, Carsten Struve, Hanne Ingmer, Anna Koza, Kosai Al-Nakeeb, Yvonne Agersø

**Affiliations:** ^1^ Research & Development, Chr. Hansen A/S, Hørsholm, Denmark; ^2^ Department of Veterinary and Animal Sciences, University of Copenhagen, Frederiksberg, Denmark

**Keywords:** antibiotic, epidemiological cut-offs, tentative ECOFFs, intrinsic resistance, antibiotic resistance, lactic acid bacteria

## Abstract

**Introduction:**

In this work, 170 strains covering 13 species from the *Lactobacillaceae* family were analyzed to determine minimal inhibitory concentration (MIC) distributions to nine antimicrobial agents, and genes potentially conferring resistance. This allows a proposal of tentative Epidemiological Cut-Offs (ECOFFs) that follows the phylogeny for interpretation of resistance in the 13 species.

**Methods:**

The 170 strains originated from different sources, geographical areas, and time periods. MICs for nine antibiotics were determined according to the ISO 10932 standard for lactobacillia and by a modified CLSI-method for *Leuconostoc* and *Pediococcus* which ensured sufficient growth. The strains were whole genome sequenced, subtyped by core genome analysis, and assessed for the presence of antibiotic resistance genes using the ResFinder and NCBI AMRFinder databases.

**Results and discussion:**

The data provide evidence that antimicrobial susceptibility follows phylogeny instead of fermentation pattern and accordingly, tentative ECOFFs were defined. For some species the tentative ECOFFs for specific antibiotics are above the cut-off values set by the European Food Safety Authority (EFSA) which are primarily defined according to fermentation pattern or at genus level. The increased tolerance for specific antibiotics observed for some species was evaluated to be innate, as only for one strain phenotypic resistance was found to be related to an acquired resistance gene. In general, more data are needed to define ECOFFs and since the number of isolates available for industrial relevant bacterial species are often limited compared to clinically relevant species, it is important; 1) that strains are unambiguously defined at species level and subtyped through core genome analysis, 2) MIC determination are performed by use of a standardized method to define species-specific MIC distributions and 3) that known antimicrobial resistance genes are determined in whole genome sequences to support the MIC determinations.

## Introduction

Antibiotic resistant organisms are present in all environments and both pathogenic and non-pathogenic bacteria encode antibiotic resistance genes (Allen et al., 2010). When non-pathogenic bacteria are included in food and feed cultures, it is a requirement that they are free of acquired antibiotic resistance genes as these may be transferred to pathogenic bacteria potentially compromising antimicrobial therapy (EFSA panel on Additives and Products or Substances used in Animal Feed (FEEDAP), 2018). Intrinsic (innate) antibiotic resistance is, however, not considered a safety concern, as it is conserved within specific species and spread clonally rather than horizontally. The major intrinsic mechanisms are absence of the antibiotic target, mutations conferring a low affinity or permeability or intrinsic genes e.g. encoding an efflux mechanism (EFSA, 2005; Cox and Wright, 2013; EFSA panel on Additives and Products or Substances used in Animal Feed (FEEDAP), 2018; Nøhr-Meldgaard et al., 2021).

To reduce the risk of transmissible antibiotic resistance genes from food and feed, the European Food Safety Authority (EFSA) provides antimicrobial microbiological cut-off values, for nine antimicrobial compounds, which are considered as highly or critically important for treatment of infections in humans (World Health Organisation (WHO), 2018). The cut-off values are a pragmatic tool for differentiating between resistant and susceptible bacterial strains within a population (EFSA panel on Additives and Products or Substances used in Animal Feed (FEEDAP), 2018). The current EFSA cut-off values are defined based on published minimal inhibitory concentration (MIC) data of industrially relevant species. However, much of the data have been generated using different methods (broth microdilution, Etest, disk diffusion and agar dilution method) and test conditions, either because the studies were performed before the ISO 10932 standard on determination of MIC for lactic acid bacteria (LAB) was published or because the proposed test conditions, such as using cation-adjusted Mueller-Hinton broth (CAMHB) with lysed horse blood for *Leuconostoc* and *Pediococcus*, does not provide the optimal growth conditions compared to the LAB susceptibility test medium (LSM) (Klare et al., 2005; International Organization for Standardization, 2010; Clinical and Laboratory Standards Institute (CLSI), 2016). Furthermore, the amount of MIC data on industrially relevant bacterial species are limited and not enough to define epidemiological cut-offs (ECOFFs), which require data from at least five separate laboratories, at least 15 values from each laboratory and at least 100 MIC values in the wild-type distribution (European Committe on Antimicrobial Susceptibility Testing (EUCAST), 2021). Due to the limited amount of MIC data on LAB, the current cut-off values for the *Lactobacillus* genus are defined primarily according to fermentation pattern e.g., obligate homofermentative, facultative heterofermentative and obligate heterofermentative, and for *Leuconostoc* and *Pediococcus* cut-off values are only defined at genus level. This is not optimal as the recommendation from EUCAST is to define cut-off values at species level, which is also supported by previous studies on industrially relevant bacterial species (Agersø et al., 2018; EFSA panel on Additives and Products or Substances used in Animal Feed (FEEDAP), 2018; European Committe on Antimicrobial Susceptibility Testing (EUCAST), 2021). Therefore, more antimicrobial susceptibility data for industrially relevant species are needed.

Traditionally, *Lactobacillus* species have been characterized based on the type of sugars fermented and the fermentation product formed and grouped as either obligate homofermentative, facultative heterofermentative or obligate heterofermentative (Salvetti et al., 2012). However, recent studies have shown that this division of *Lactobacillus* species is obsolete as it does not follow phylogeny and in 2020, a major taxonomic revision of the *Lactobacillus* genus was performed, which resulted in the splitting of the *Lactobacillus* genus into 25 genera and the inclusion of the *Leuconostoc* genera in the *Lactobacillaceae* family, which already included *Pediococcus* (Salvetti et al., 2012; Zheng et al., 2015; Duar et al., 2017; Zheng et al., 2020). As a consequence of the taxonomic revision, the MIC of species belonging to different genera, such as *Lentilactobacillus parabuchneri and Limosilactobacillus fermentum* should be evaluated using the same cut-off values, namely the *Lactobacillus* obligate heterofermentative cut-off values (EFSA panel on Additives and Products or Substances used in Animal Feed (FEEDAP), 2018). This illustrates the need for updated microbiological cut-off values for *Lactobacillaceae* that follows phylogeny instead of fermentation patterns.

*Leuconostoc* species are important for the production of fermented dairy products (Cardamone et al., 2011) and the majority of published microbiological susceptibility data are on the industrially relevant species *Leuconostoc mesenteroides* and *Leuconostoc pseudomesenteroides.* However, several different methods and test conditions have been used, wherefore data generated using standardized test conditions are needed (Swenson et al., 1990; Katla et al., 2001; Casado Muñoz M del et al., 2014; Basbülbül et al., 2015; Jeong and Lee, 2015; Flórez et al., 2016). Recently, the *L. pseudomesenteroides* species were divided into two species, namely *L. pseudomesenteroides* and the novel *Leuconostoc falkenbergense* species (Wu and Gu, 2021). However, *L. falkenbergense* and *L. pseudomesenteroides* are more closely related to each other than to other *Leuconostoc* species including *L. mesenteroides* (Wu and Gu, 2021).

Strains of the species *P. acidilactici* and *P. pentosaceus* are frequently used for cheese production, but are also used as probiotics, and meat and vegetables fermentations as they produce characteristic flavor and improve hygienic quality and extend shelf life due to the production of bacteriocins (Stiles, 1996; Holzapfel et al., 1998; Beresford et al., 2001). Due to their important role in fermentation, most of the published antimicrobial susceptibility data for *Pediococcus* are for the *P. acidilactici* and *P. pentosaceus* species; however, different methods and test conditions have been used which can affect the MIC values (Swenson et al., 1990; Danielsen et al., 2007; Klare et al., 2007; Muñoz-Atienza et al., 2013).

In the present study, tentative ECOFFs will be defined for 13 LAB species and evaluated against the currently available EFSA cut-off values which are primarily defined according to fermentation pattern or at genus level. Our results show that cut-off values should be based on phylogenetic relatedness rather than fermentation pattern and at species rather than genus level. This will improve the interpretation criteria for antimicrobial susceptibility for these species.

## Materials and methods

### Bacterial strains

One hundred and seventy strains, including the specific type strains, belonging to 13 species were included in the study (**Table S1**). The strains were obtained from Chr. Hansen’s Culture collection (CHCC), where they were stored at -80°C. The strains cover different geographic areas, sources and timepoints (**Table S1**).

### Genomic DNA extractions, library preparation and QC for *de novo* short read (Illumina) whole genome sequencing

Genomic DNA for *de novo* short read WGS was extracted from bacterial cell pellets harvested from 1 mL of overnight culture normalized to OD_600 _= 1. Clean Blood & Tissue DNA Kit (NACBT-D0384) (Clean NA, The Netherlands) was used and manufactures protocol was modified. The extraction method was automated and performed on Biomek i5 liquid handler (Beckman Coulter, USA). Modifications to the manufactures protocol: cell pellets were resuspended in 200 µL of pre-lysis buffer (PBS, 20 mg/mL lysozyme, 50 U/mutanolysin, 100 mg/mL RNase A) instead of the Tissue Lysis buffer supplied in the kit.

Genomic libraries were generated for most of the strains using modified Kapa Hyper Plus Library Preparation Kit (Roche, Switzerland) on Biomek i5 Liquid Handler (Beckman Coulter, USA). 150 ng of genomic DNA diluted in 15 µL EB buffer (Tris-Cl, pH 8.0) was used in the half-volume reaction mixes for fragmentation, end-repair/A-tailing, ligation, and final amplification. 0.1 mM conditioning solution was added to fragmentation mix and fragmentation time was optimized to 10 minutes. 5 µL of 1 µM Kapa Dual-Indexed adapter (Roche, Switzerland) was used during adapter ligation step. 10 µL of the adapter-modified DNA fragments were enriched by 8-cycle PCR. Clean NGS beads (Clean NA, The Netherlands) were used for two post-ligation and two post-amplification clean-ups to purify fragments at average size between 450 to 550 bp.

For about 15 of the strains, genomic libraries were generated using NEBNext^®^ Ultra™ II FS DNA Library Prep Kit for Illumina^®^ with NEBNext Multiplex Oligos for Illumina (Unique Dual Index UMI Adaptors DNA Set 1), (New England Biolabs Inc., USA) on Biomek i5 Liquid Handler (Beckman Coulter, USA). 200 ng of genomic DNA diluted in 15 µL EB buffer (Tris-Cl, pH 8.0) was used in the half-volume reaction mixes for fragmentation, end-repair/A-tailing, ligation, and final amplification. Fragmentation time was optimized to 8 minutes. 5 µL of 2.5 µM NEBNext Multiplex Oligos for Illumina (Unique Dual Index UMI Adaptors DNA Set 1), (New England Biolabs Inc., USA) was used during adapter ligation step. 10 µL of the adapter-modified DNA fragments were enriched by 9-cycle PCR. Clean NGS beads (Clean NA, The Netherlands) were used for double-sided post ligation size selection and one post-amplification clean-up to purify fragments at average size between 450 to 550 bp.

Concentration of genomic DNA and dsDNA libraries were measured by QubitFlex^®^ Fluorimeter using Qubit dsDNA Broad range and Qubit 1x dsDNA HS assays (Thermo Fisher Scientific, USA), respectively. Average dsDNA library size distribution was determined using the Agilent HS NGS Fragment (1-6000 bp) kit on the Agilent Fragment Analyzer (Agilent Technologies, USA). Libraries were normalized and pooled in the normalization buffer (10 mM Tris-Cl, pH 8.0, 0.05% Tween 20) to the final concentration of 10 nM.

For most of the strains, denaturated in 0.2N NaOH, 10 pM pool of libraries in 600 μL ice-cold HT1 buffer was loaded onto the flow cell provided in the MiSeq Reagent kit v3 (600 cycles) and sequenced on a MiSeq platform (Illumina Inc., San Diego, USA) with a paired-end protocol and read lengths of 301 nt.

For about 15 of the strains, denaturated in 0.2N NaOH, 1 pM pool of libraries in 1300 μL ice-cold HT1 buffer was loaded onto the flow cell provided in the NextSeq Reagent Mid Output (300 cycles) and sequenced on a NextSeq platform (Illumina, USA) with a paired-end protocol and read lengths of 151 nt.

### Genome assembly

All processing of the short reads was done in either CLC Genomics Server version 20.0.5 or CLC Genomics Workbench version 20.0.5.

The short reads were mapped with default parameters to the reference sequence of the phage Phi X 174 using the tool “Map reads to reference”. Unmapped reads from the mapping were trimmed for quality using the PHRED score 23 as the threshold and with the non-default parameter of discarding reads that were less than 50 base pairs long using the tool “Trim Sequences”.

The trimmed reads were *de novo* assembled with default parameters except for the minimum contig length which was set to 350 base pairs using the tool “*De Novo* Assembly”. Afterwards, a decontamination step was performed where contigs with low depth of coverage were removed using a custom plugin written by Qiagen. The decontamination step first removes all contigs where the average depth of coverage is below 15X and afterwards removes all contigs where the depth of coverage is below 25% of the median average depth of coverage for the entire genome assembly.

Gene calling of the filtered contigs was done with Prodigal version 2.6.3 using the default parameters. Finally, the genome assemblies with annotated genes were functionally annotated with BLAST against a local annotation database using a custom plugin written by Qiagen.

### Species identification

Species identification was done in an automated flow by either blasting of the WGS against 16S, rpoA sequences of type strain, or average nucleotide identity in CLC Genomics Workbench version 20 (Qiagen Bioinformatics, Aarhus, Denmark). The species identification was further confirmed using core genome analysis. In brief, the genomes, either fully assembled or contigs were annotated by Prokka, which annotates genomes through the use of different tools including Prodigal (coding sequences), RNAmmer (Ribosomal RNA genes), Aragorn (Transfer RNA genes), SignalP (Signal leader peptides) and Infernal (Non-coding RNA) (Seemann, 2014). Prokka annotation is a requirement for using Roary, since the.gff file (file containing sequences and annotations) provided by Prokka is used by Roary to create a multi-FASTA alignment of all the core genes (Page et al., 2015). Roary was set to perform nucleotide alignment using MAFFT and a BLASTP percentage identity between 80-100%, depending on species (Katoh, 2002). FastTree was used to produce an approximately-maximum-likelihood phylogenetic tree from the core gene alignment file, which was visualized by MEGA X (Price et al., 2009; Price et al., 2010; Kumar et al., 2018).

### Antimicrobial susceptibility testing

The MIC of nine antimicrobial agents was determined by use of broth microdilution, where the MIC is the lowest concentration of the antimicrobial that inhibits bacterial growth (Adimpong et al., 2012). All species were tested in LSM medium, which consist of 10% Iso-Sensitest (IST) broth and 90% MRS (De Man, Rogosa, Sharpe) medium both from Oxoid.

For the *Lactobacillus* species and species formerly belonging to the *Lactobacillus* genus, the strains were tested as recommended by the ISO 10932 standard (International Organization for Standardization, 2010), *P. acidilactici* was tested by use of the CLSI method (LSM media, 35°C, aerobic with film), while *P. pentosaceus* was tested by the use of a modified CLSI method (LSM, 30°C, aerobic with a lid). *L. mesenteroides, L. falkenbergense* and *L. pseudomesenteroides* were also tested by use of a modified CLSI method (LSM, 30°C, aerobic with film). MIC was read at both 20 and 24 hours for the *Pediococcus* genus and at 24 and 48 hours for the *Leuconostoc* genus.

*L. plantarum* ATCC 14917 and *L. paracasei* ATCC 334 were included for quality control using quality control ranges reported in the ISO 10932 standard (International Organization for Standardization, 2010). For 10 out of 40 *Leuconostoc* strains (3 media batches) the quality control strain *L. plantarum* exhibited ampicillin and clindamycin MIC one 2-fold below the accepted range, however when the quality control strain *L. paracasei* was tested with the same medium batch it was within the accepted range.

All tests were performed in duplicates in a customized Sensititre panel from Thermo Fisher Scientific. Nine antimicrobial agents are included in the customized Sensititre panel: ampicillin 0.03-16 mg/L, chloramphenicol 0.5-54 mg/L, clindamycin 0.03-32 mg/L, erythromycin 0.015-16 mg/L, gentamycin 0.25-128 mg/L, kanamycin 1-1024 mg/L, streptomycin 1-256 mg/L, tetracycline 0.12-64 mg/L and vancomycin 0.12-16 mg/L. Retesting was performed if the duplicates varied more than one 2-fold dilution for one or more antimicrobial agents. The results were accepted if they varied by three or fewer two-fold concentrations as previously described being within the technical variation for MIC broth dilution methods (Clinical and Laboratory Standards Institute (CLSI), 2018).

If the MIC value differed one 2-fold between the duplicates, the highest MIC was reported. All strains were streaked on blood agar plates to ensure that the samples were pure.

To compare the results from the customized Sensititre panel and the discontinued VetMIC panels Lact-1 and Lact-2 (SVA, Uppsala, Sweden), MIC data from 2012-2019 was compared for 25 strains on both MIC panels using the same method.

### Epidemiological cut-off values for differentiation of susceptible (wildtype) and resistant (non-wildtype) populations

For each species-antimicrobial combination, MIC distributions were determined and from this tentative ECOFFs were defined together with MIC_50_ and MIC_90_ (MICs inhibiting 50% and 90% of the strains, respectively). ECOFFs is defined according to guidelines from the European Committee on Antimicrobial Susceptibility Testing (EUCAST) (Turnidge et al., 2006; European Committe on Antimicrobial Susceptibility Testing (EUCAST), 2021), which state that the population with MIC at or below the ECOFF are susceptible (wildtype) and therefore also devoid of acquired resistance mechanisms and/or mutations leading to resistance (European Committe on Antimicrobial Susceptibility Testing (EUCAST), 2021).

Moreover, according to EUCAST the intrinsic (or wildtype) population is also characterized by the absence of acquired resistance mechanisms and/or mutations leading to resistance (European Committe on Antimicrobial Susceptibility Testing (EUCAST), 2021). The data were also evaluated with the interpretation criteria defined by EFSA for *Bacillus* ssp. (EFSA panel on Additives and Products or Substances used in Animal Feed (FEEDAP), 2018).

### Detection of known antimicrobial resistance genes and comparison with phenotype

The presence of genes with identity to known antimicrobial resistance genes, in all the strain genomes, was assessed using ResFinder (Zankari et al., 2012) (nucleotide) and NCBI AMRFinderPlus (Feldgarden et al., 2019) (amino acid). Both databases were downloaded and imported into CLC Genomics Workbench 20.0.5. ResFinder was imported on 20 April 2021 and AMRFinderPlus on 27 April 2021. The assembled contigs of each strain were joined using the join function in CLC. The joined contigs were screened for resistance genes against the Resfinder database using BLASTn with a minimum word size of 11 and maximum E-value of 1.0E-10 and AMRFinderPlus using BLASTn with a minimum word size of 3 and a maximum E-value of 1.0E-50.

EFSA require that sequences with at least 80% identity and 70% coverage to known antimicrobial resistance genes should be reported. In the case two or more fragments covering less than 70% length of the subject sequence with at least 80% identity to the same antimicrobial resistance gene are detected these should be reported, and it should be checked whether the full gene is present (European Food Safety Authority, 2021). The same criteria were used in this study.

## Results and discussion

### Comparison of MIC measured by VetMIC and Sensititre panels

The ISO 10932 standard on antimicrobial susceptibility testing of industrially used species suggest using VetMIC panels (SVA, Uppsala, Sweden) for MIC determination (International Organization for Standardization, 2010). However, as VetMIC panels have been discontinued by the provider alternative panels need to be evaluated. Therefore, MIC for 25 strains covering nine of the 13 species included in the study were measured using the VetMIC and the customized Sensititre panels (**Table S2**) to ensure comparable results are obtained. The MIC for specific strain-antimicrobial agents combinations varied less than three 2-fold dilutions for the VetMIC and Sensititre panels, which is described as the technical variation acceptable for the broth microdilution method (Clinical and Laboratory Standards Institute (CLSI), 2018). Therefore, the results obtained from the two panels are comparable when the strains are tested with the same conditions and the customized Sensititre panels can replace the VetMIC panels.

### Included strains and grouping based on phylogenetic relatedness

In the present study, 170 strains belonging to 13 species, including the type strains were obtained from Chr. Hansen’s Culture collection. The strains were epidemiologically unrelated and have been isolated from different geographic areas, sources and timepoints (**Table S1**). The criteria for including the specific species were 1) the current microbiological cut-offs are only defined at genus level (*Pediococcus* and *Leuconostoc*) or 2) the current microbiological cut-offs are defined based on fermentation groups and novel genera have been defined due to the recent *Lactobacillaceae* taxonomic revision (*Lactobacillus, Lactilactobacillus, Lentilactobacillus, Ligilactobacillus, Limosilactobacillus*) (Zheng et al., 2020). The included *Lactobacillus* species (*L. delbrueckii, L. gasseri, L. paragasseri, L. helveticus*) were chosen as a broad representation of the *Lactobacillus* genus (Zheng et al., 2020).

Core genome analysis was performed for each species to ensure that the included strains were phylogenetically different and based on this, 32 strains were excluded, which resulted in 170 strains included in the study.

**Figure 1:**
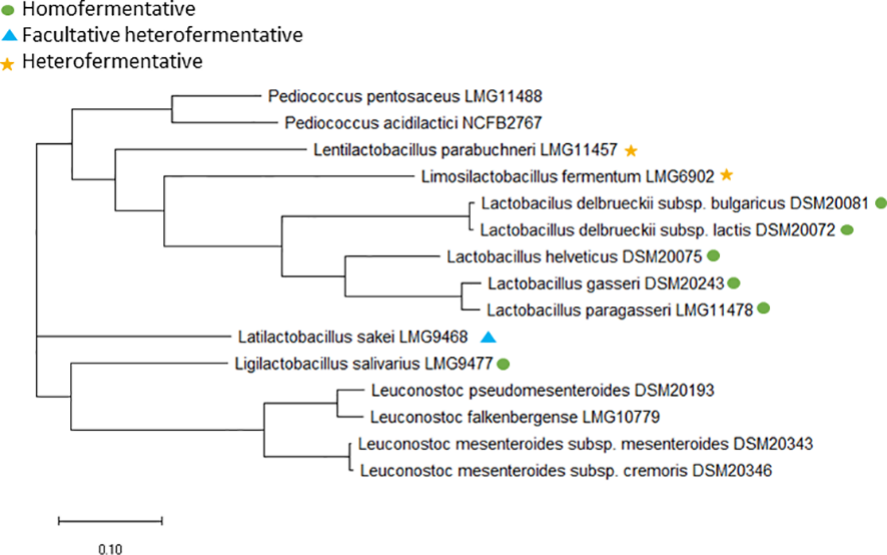
Core genome phylogenetic tree based on 65 core genes for the type strains of the included species. *Lactobacillus, Limosilactobacillus, Lentilactobacillus, Lactilactobacillus and Ligilactobacillus* species are either marked with a green dot if obligate homofermentative, a blue triangle if facultative heterofermentative or a yellow star if obligate heterofermentative. NCBI accession no.: LMG11488, SAMN33225762; NCFB2767, SAMN33225763; LMG11457, SAMN33225768; LMG6902, SAMN33225764; DSM20081, SAMN33225756; DSM20072, CP022988.1; DSM20075, SAMN33225755; DSM20243, SAMN33225757; LMG11478, SAMN33225767; LMG9468, SAMN33225759; LMG9477, SAMN33225758; DSM20193, SAMN33225760; LMG10779, SAMN33225766; DSM20343, SAMN33225765; DSM20346; SAMN33225761.

Furthermore, core genome analysis of the type strains from the 13 included species was performed (**Figure 1**) to determine whether some of the species are so closely related that combined tentative ECOFFs can be defined and to verify that phylogeny and fermentation patterns is not related. The analysis shows that the phylogenetic grouping does not follow the fermentation pattern for *Lactobacillus* species and species previously belonging to the *Lactobacillus* genus, which is in agreement with previous studies (Zheng et al., 2015; Zheng et al., 2020) (**Figure 1**). This supports that *Lactobacillaceae* tentative ECOFFs should be defined according to phylogeny instead of fermentation patterns. Species specific tentative ECOFFs will therefore be defined for all the included *Lactobacillus, Lactilactobacillus, Lentilactobacillus, Ligilactobacillus* and *Limosilactobacillus* species, expect the phylogenetically closely related species *Lactobacillus gasseri* and *Lactobacillus paragasseri* (Tanizawa et al., 2018; Zheng et al., 2020) (**Figure 1**) for which the MIC distributions for the eight examined agents were overlapping.

**Figure 2:**
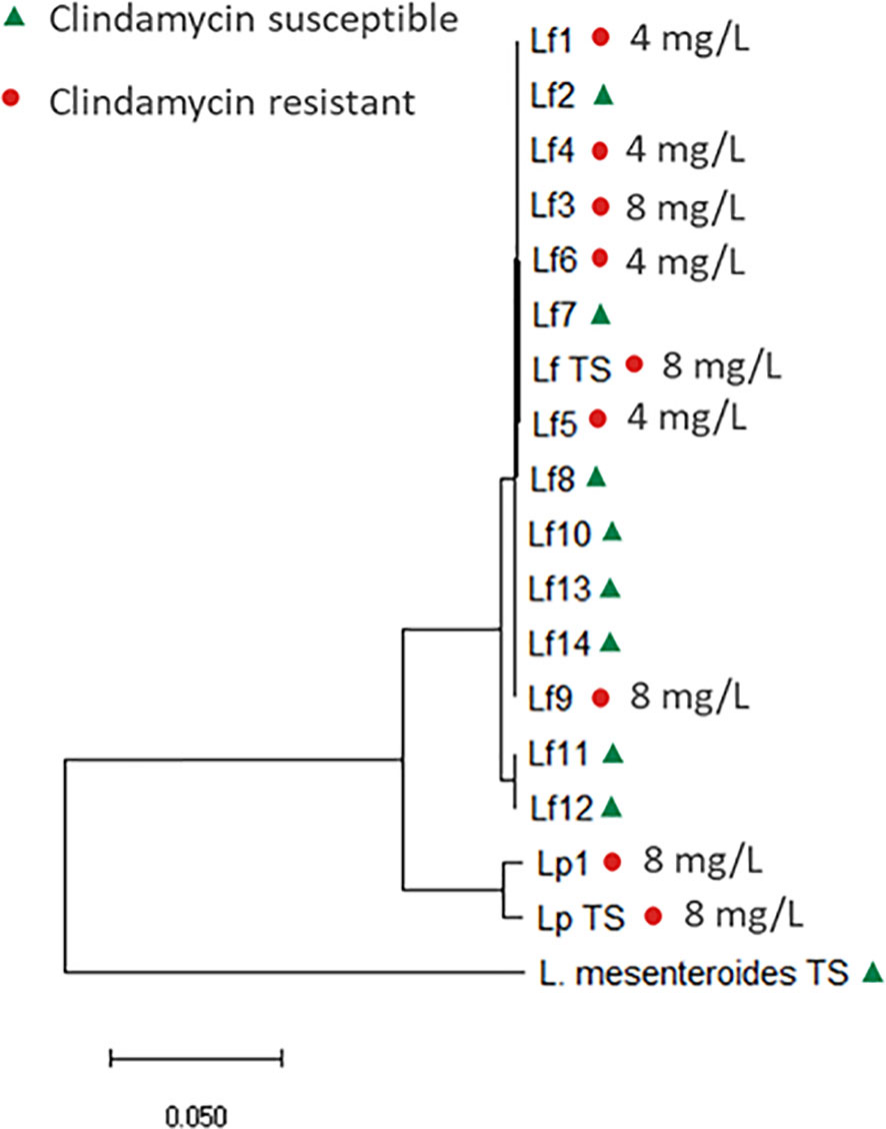
Core genome phylogenetic tree based on 592 core genes, including the 15 *L. falkenbergense* strains, named Lf1-14 and Lf TS (type strain, LMG10779) and the two *L. pseudomesenteroides* strains named Lp1 and Lp TS (type strain, DSM20193). The tree is rooted with the *L. mesenteroides* type strain (DSM20346). Clindamycin susceptible strains are marked with a triangle (green) and resistant strains with a dot (red).

For *Leuconostoc*, EFSA have defined microbiological cut-off values at genus level (EFSA panel on Additives and Products or Substances used in Animal Feed (FEEDAP), 2018). Two *Leuconostoc* species, *L. mesenteroides* and *L. pseudomesenteroides* was initially included in the present study; however, recently, the *L. pseudomesenteroides* species was divided into two species: *L. pseudomesenteroides* and the novel species *L. falkenbergense* (Wu and Gu, 2021). Core genome analysis revealed that all but two of the included *L. pseudomesentoides* strains belong to the *L. falkenbergense* species (**Figure 2**). As *L. falkenbergense* and *L. pseudomesenteroides* are very closely related both based on 16S rRNA sequence (Wu and Gu, 2021) and core genome analysis (**Figure 1**), tentative ECOFFs will be defined for the *L. falkebergense*/*L. pseudomesenteroides* group while tentative ECOFFs will be defined individually for *L. mesenteroides.*

Overall, the strains were epidemiologically unrelated and genetically diverse, so the strain collection displays a good representation of most of the included species, although the number of isolates were limited. Another limitation is that the MIC analysis was performed in only one laboratory and not in several, the ECOFFs defined in this study are therefore tentative.

### Comparison of MIC

### Obligate homofermentative

The MIC range of the four homofermentative *Lactobacillus* species (*L. delbrueckii, L. gasseri/paragasseri, L. helveticus*) was compared to the *Lactobacillus* obligate homofermentative microbiological cut-off values provided by EFSA (**Table 1**) (EFSA panel on Additives and Products or Substances used in Animal Feed (FEEDAP), 2018; Zheng et al., 2020). Overall, these species exhibit different MIC distributions for all nine tested antimicrobial agents illustrating the need for tentative ECOFFs that follow phylogeny (**Table 1**).

**Table 1:** MIC distribution and tentative ECOFFs for nine antimicrobial agents for *Lactobacillus* obligate homofermentative species.

Antimicrobial agent	Species	Distribution (%) of MICs																		Tentative	MIC50	MIC90
		0.0075	0.015	0.03	0.06	0.12	0.25	0.5	1	2	4	8	16	32	64	128	256	512	1024	ECOFF		
Ampicillin	*L. delbrueckii* (28) *L. gasseri* (7)*/L. paragasseri* (9) *Lactobacillus helveticus* (7)		18		39	36	7													0.25	0.06	0.21
						12	50	38												0.5	0.25	0.5
							14	16												0.5	0.5	0.5
Chloramphenicol	*L. delbrueckii* (28) *L. gasseri* (7)*/L. paragasseri* (9) *Lactobacillus helveticus* (7)								4	18	75	4								8	4	4
											38	62								8	8	8
											100									4	4	4
Clindamycin	*L. delbrueckii* (28) *L. gasseri* (7)*/L. paragasseri* (9) *Lactobacillus helveticus* (7)		21		50	21	4	4												0.5	0.06	0.12
							19	13	6	6	31	25								8	4	8
								29	14	57										2	2	2
Erythromycin	*L. delbrueckii* (28) *L. gasseri* (7)*/L. paragasseri* (9) *Lactobacillus helveticus* (7)	21		29	39	11														0.12	0.03	0.06
					31	56	13													0.25	0.12	0.12
				29	71															0.06	0.06	0.06
Gentamycin	*L. delbrueckii* (28) *L. gasseri* (7)*/L. paragasseri* (9) *Lactobacillus helveticus* (7)					4		14	25	25	18	14								8	2	8
										56	38	6								8	4	4
								29	43	29										2	1	2
Kanamycin	*L. delbrueckii* (28) *L. gasseri* (7)*/L. paragasseri* (9) *Lactobacillus helveticus* (7)							7		7	18	11	25	4	18	11				128	8	32
												13	6	31	38	13				128	64	128
												57	29	14						32	8	32
Streptomycin	*L. delbrueckii* (28) *L. gasseri* (7)*/L. paragasseri* (9) *Lactobacillus helveticus* (7)							4		14	11	14	25	4						32	8	32
											31	56	6	6						32	8	16
								43		57										2	2	2
Tetracycline	*L. delbrueckii* (28) *L. gasseri* (7)*/L. paragasseri* (9) *Lactobacillus helveticus* (7)						7	4	32	50	7									4	2	4
										13	69	19								8	4	8
										43	57									4	4	4
Vancomycin	*L. delbrueckii* (28) *L. gasseri* (7)*/L. paragasseri* (9) *Lactobacillus helveticus* (7)						21	75	4											1	0.5	1
									81	19										2	1	2
									86	14										2	1	2

MIC is compared to the Lactobacillus obligate homofermentative microbiological cut-off values by EFSA (vertical line). The white area shows the tested concentration of the specific antimicrobials and the grey area shows the concentration of the specific antimicrobials not tested.

None of the *Lactobacillus* species (*L. gasseri/paragasseri, L. helveticus, L. delbrueckii*) exhibit vancomycin MIC above the *Lactobacillus* obligate homofermentative cut-off value of 2 mg/L, in accordance with previous findings (Delgado et al., 2005; Zhang et al., 2018).

It is generally reported in the scientific literature that *Lactobacillus* spp. exhibits a high tolerance towards aminoglycosides and especially kanamycin as an intrinsic property of the genus (Danielsen and Wind, 2003; Mathur and Singh, 2005; Mayrhofer et al., 2010; Nawaz et al., 2011; Adimpong et al., 2012). In the present study, both *L. gasseri*/*paragasseri* and *L. delbrueckii* exhibit a kanamycin MIC range up to 128 mg/L (8-128 mg/L and ≤ 1-128 mg/L, respectively) and most of the population showed MICs above the EFSA cut-off value at 16 mg/L. This is in accordance with previous studies using broth microdilution method and test conditions as recommended in the ISO 10932 standard (International Organization for Standardization, 2010; Mayrhofer et al., 2010; Nawaz et al., 2011) (**Table 1**). Based on the included strains, *L. delbrueckii* subsp. *lactis* exhibit one 2-fold dilution higher kanamycin MIC range than the *L. delbrueckii* subsp. *bulgaricus* strains, but both subspecies exhibit a broad kanamycin MIC range. Furthermore, the *L. delbrueckii* subsp. *bulgaricus* type strain exhibit kanamycin MIC of 64 mg/L, while *L. delbrueckii* subsp. *lactis* type strain exhibit kanamycin MIC of 4 mg/L. This indicates that reduced kanamycin susceptibility is not only related to a specific subspecies; however, more strains belonging to the two subspecies need to be examined to evaluate this.

In contrast, *L. helveticus* exhibit a kanamycin MIC range of 8-32 mg/L, suggesting that innate tolerance to kanamycin is species specific and tentative ECOFFs should be defined according to phylogeny. Furthermore, *L. helveticus* exhibit streptomycin (and gentamycin) MIC values markedly below the current cut-off at 16 mg/L, as previously shown (Klare et al., 2007) showing that aminoglycoside susceptibility differ within species belonging to the *Lactobacillus* genus and obligate homofermentative species.

The current erythromycin EFSA cut-off is 1 mg/L, which is two-four 2-fold dilutions higher than the observed MIC distributions for the four *Lactobacillus* species (**Table 1**), in accordance with previous findings (Klare et al., 2007; Nawaz et al., 2011; EFSA panel on Additives and Products or Substances used in Animal Feed (FEEDAP), 2018). This illustrates that the current EFSA cut-off values also can be too high for specific species and should be adjusted to divide the wild-type population from strains potentially coding for acquired resistance genes.

### Facultative heterofermentative

As recommended by EFSA, the MIC ranges of *L. sakei* and the homofermentative *Ligilactobacillus salivarius* species were compared to the *Lactobacillus* facultative heterofermentative microbiological cut-off values (**Table 2**) (EFSA panel on Additives and Products or Substances used in Animal Feed (FEEDAP), 2018; Zheng et al., 2020).

**Table 2:** MIC distribution and tentative ECOFFs for nine antimicrobial agents for *Ligilactobacillus salivarius and Lactilactobacillus sakei*.

Antimicrobial	Species	Distributions (%) of MICs																		Tentative		
		0.0075	0.015	0.03	0.06	0.12	0.25	0.5	1	2	4	8	16	32	64	128	256	512	1024	ECOFF	MIC50	MIC90
Ampicillin	*L. salivarius* (12)	* *					8	92												0.5	0.5	0.5
	*L. sakei* (10)								10	50	40									4	2	4
Chloramphenicol	*L. salivarius* (12)	* *								17	50	33								8	4	8
	*L. sakei* (10)										100									4	4	4
Clindamycin	*L. salivarius* (12)	* *	25		8		42	17	8											1	0.25	0.5
	*L. sakei* (10)		20		10	10		20	20	20										2	0.5	2
Erythromycin	*L. salivarius* (12)	* *				8	17	75												0.5	0.5	0.5
	*L. sakei* (10)						80	20												0.5	0.25	0.5
Gentamycin	*L. salivarius* (12)	* *								8	17	50	25							16	8	16
	*L. sakei* (10)										40	50	1							16	8	16
Kanamycin	*L. salivarius* (12)	* *													8	33	25	33		512	256	512
	*L. sakei* (10)											20	30	50						32	32	32
Streptomycin	*L. salivarius* (12)	* *											8	8	50	33				128	64	128
	*L. sakei* (10)													10	30	60				128	128	128
Tetracycline	*L. salivarius* (12)	* *							17	25	50	8								8	4	8
	*L. sakei* (10)									10	80					10				4	4	4
Vancomycin	*L. salivarius* (12)	* *												100						>16	>16	>16
	*L. sakei* (10)													100						>16	>16	>16

MIC is compared to the Lactobacillus facultative heterofermentative microbiological cut-off values by EFSA (vertical line). The white area shows the tested concentration of the specific antimicrobial and the grey area shows the concentration of the specific antimicrobial not tested.

Previous studies have shown that *L. salivarius* exhibits elevated kanamycin MIC (Nawaz et al., 2011; Adimpong et al., 2012; Stefańska et al., 2021), which is also observed in the present study, where 92% of the *L. salivarius* population exhibit kanamycin MICs above the current cut-off (64 mg/L), with a MIC range of 64-512 mg/L (**Table 2**). Since the whole population exhibit an elevated kanamycin MIC range it can be considered as an inherent trait of the species and the kanamycin tentative ECOFFs should be adjusted to reflect this. In contrast, *L. sakei* exhibit a lower kanamycin MIC range of 8-32 mg/L.

For ampicillin and clindamycin, the examined *L. salivarius* strains exhibit ampicillin and clindamycin MIC distributions two or three 2-fold dilutions below the current cut-off, suggesting the need for adjusting the cut-off values for these two antimicrobial agents.

Both *L. salivarius* and *L. sakei* are resistant to vancomycin (MIC >16 mg/L), which previously have been reported for several species of LAB (Swenson et al., 1990; Klare et al., 2007; Muñoz-Atienza et al., 2013; Flórez et al., 2016; Zhang et al., 2018). This is related to the presence of D-Ala-D-lactate in the peptidoglycan of these species rather than a D-Ala-D-Ala dipeptide (Flórez et al., 2016).

Of the tested *L. sakei* strains, 60% were found to exhibit streptomycin MIC values of 128 mg/L, which is above the current cut-off of 64 mg/L, indicating that the cut-off should be adjusted.

One *L. sakei* strain (Accession number JANRGY000000000) showed a tetracycline MIC value above 64 mg/L, which is more than four 2-fold dilutions above the rest of the population, which showed a MIC distribution below the EFSA cut-off value (**Table 2**). Genomic analysis revealed that the strain encodes a ribosomal protection *tet*(M) gene with 100% nucleotide identity and 100% coverage to a gene from *Staphylococcus aureus* (accession number FN433596) and also a truncated variant of a gene with high identity (99.55% nucleotide identity and 81% coverage) to *tet*(L) gene from a *Bacillus* sp. plasmid encoding a an MFS efflux resistance pump (accession number HM235948). A previous study has reported a *L. sakei* strain encoding both a chromosomally located transposon-associated *tet*(M) gene (accession number EF605269) and a plasmid-carried *tet*(L) gene (accession number EF605268), with high identity to a plasmid-encoded *tet*(L) gene from *Paenibacillus larvae* (Murray and Aronstein, 2006; Ammor et al., 2008). The *tet*(M) and *tet*(L) encoded by the *L. sakei* strain (Accession number JANRGY000000000) in the present study are surrounded by genes both originating from EF605269, EF605268 and a *L. sakei* plasmid (CP025207) (**Figure S1**), suggesting it have been acquired (Davray et al., 2021).

### Obligate heterofermentative

For the two heterofermentative species (*Lentilactobacillus parabuchneri* and *Limosilactobacillus fermentum*), the MIC ranges are compared to the EFSA microbiological cut-off values for *Lactobacillus* obligate heterofermentative (**Table S3**). The two species exhibit different MIC distributions toward the tested antimicrobial agents, which was expected as they belong to different genera, again supporting the need for defining cut-off values that follows phylogeny rather than fermentation pattern.

All the tested *L. parabuchneri* strains exhibit tetracycline MIC above the current cut-off value at 8 mg/L, with a MIC range of 16-64 mg/L (**Table S3**), in accordance with previous findings (Nawaz et al., 2011). A previous study has found that the species belonging to the novel *Lentilactobacillus* genus all exhibit tetracycline MIC above the EFSA cut-off of 8 mg/L, suggesting that the EFSA recommended tetracycline cut-off value for *L. buchneri* at 128 mg/L is also applicable to all the species belonging to *Lentilactobacillus;* however, more data on the individual species are needed to conclude this (Feichtinger et al., 2016; EFSA panel on Additives and Products or Substances used in Animal Feed (FEEDAP), 2018).

Even though studies have shown that some *Lactobacillus* species exhibit a high tolerance toward aminoglycosides (Turnidge et al., 2006; Price et al., 2009; Price et al., 2010; Zankari et al., 2012; Kumar et al., 2018), both *L. parabucneri* and *L. fermentum* exhibit gentamycin MIC two-four 2-fold dilutions below the current cut-off of 16 mg/L, in accordance with previous studies (Klare et al., 2007; Nawaz et al., 2011) again illustrating that aminoglycoside resistance pattern is species specific.

All the tested *L. fermentum* strains exhibit chloramphenicol MIC above the current cut-off at 4 mg/L, which is in accordance with previous findings (Klein, 2011).

### Pediococcus

MIC values were measured for strains belonging to *P. acidilactici* and *P. pentosaceus* (**Table 3**). CLSI recommend using CAMHB with lysed horse blood when assessing antimicrobial susceptibility for *Pediococcus*. However, as a study has shown that LSM provide better growth of *Pediococcus*, LSM was used in the present study. Furthermore, CLSI recommend reading MIC between 20-24 hours to ensure good growth, however, a standardized MIC reading time point is preferable to correctly compare MIC values. In this study, the MIC was read both after 20 and 24 hours incubation and all the included strains were found to show adequate growth in the control wells at 20 hours. Furthermore, the MIC values did not increase more than one 2-fold between the 20 hours and 24 hours reading. We therefore recommend recording MIC at 20 hours for *Pediococcus* species, since adequate growth in the control wells was observed for alle the tested strains at this timepoint and further growth could potentially lead to overestimation of the MIC values.

**Table 3:** MIC distribution and tentative ECOFFs for nine antimicrobial agents for the *Pediococcus* species.

Antimicrobial	Species	Distribution (%) of MICs																		Tentative		
		0.0075	0.015	0.03	0.06	0.12	0.25	0.5	1	2	4	8	16	32	64	128	256	512	1024	ECOFF	MIC50	MIC90
Ampicillin	*P. acidilactici* (21)								5	48	48									4	2	4
	*P. pentosaceus* (21)									10	90									4	4	4
Chloramphenicol	*P. acidilactici* (21)										100									4	4	4
	*P. pentosaceus* (21)									38	57	5								8	4	4
Clindamycin	*P. acidilactici* (21)		81		19															0.06	≤0.03	0.06
	*P. pentosaceus* (21)		72		14	14														0.12	≤0.03	0.12
Erythromycin	*P. acidilactici* (21)				5	43	47	5												0.5	0.25	0.25
	*P. pentosaceus* (21)					62	33	5												0.5	0.12	0.25
Gentamycin	*P. acidilactici* (21)									9	81	10								8	4	4
	*P. pentosaceus* (21)								9	29	43	14	5							16	4	8
Kanamycin	*P. acidilactici* (21)													5	19	76				128	128	128
	*P. pentosaceus* (21)											5		14	48	33				128	64	128
Streptomycin	*P. acidilactici* (21)													14	86					64	64	64
	*P. pentosaceus* (21)													10	57	33				128	64	128
Tetracycline	*P. acidilactici* (21)											57	43							16	8	16
	*P. pentosaceus* (21)											52	48							16	8	16
Vancomycin	*P. acidilactici* (21)													100							>16	>16
	*P. pentosaceus* (21)													100							>16	>16

MIC is compared to the Pediococcus microbiological cut-off values by EFSA (vertical line).The white area shows the tested concentration of the specific antimicrobials and the grey area shows the concentration of the specific antimicrobials not tested.

For both *P. acidilactici* and *P. pentosaceus*, trailing endpoints were observed for tetracycline, which are defined as a gradual fading of growth over two-three wells. This phenomenon have been described for Gram-positive cocci when tested against bacteriostatic antimicrobial agents such as tetracycline (EUCAST, 2022). The tetracycline MIC was determined as the first well with significant growth inhibition compared to the control wells as recommended by EUCAST (2022).

Overall, *P. acidilactici* and *P. pentosaceus* (**Table 3**) exhibit similar MIC distributions for the tested antimicrobial agents. The MIC ranges for chloramphenicol, kanamycin, streptomycin, and tetracycline were found to be one-two 2-fold dilutions higher than the current microbiological cut-offs provided by EFSA (**Table 3**), which could be explained by the different methods and test conditions used to measure MIC for *Pediococcus* and that the LSM medium provide better growth of *Pediococcus* compared to CAMHB with lysed horse blood (Swenson et al., 1990; Tankovic et al., 1993; Klare et al., 2005; Rojo-Bezares et al., 2006; Danielsen et al., 2007; Klare et al., 2007; Muñoz-Atienza et al., 2013; Basbülbül et al., 2015). This supports the need for standardized methods and test conditions when measuring MIC for defining tentative ECOFFs.

### Leuconostoc

MIC was measured for strains belonging to *L. falkebergense*/*L. pseudomesenteroides* and *L. mesenteroides* (**Table 4**). CLSI recommend reading MIC between 24 and 48 hours for the *Leuconostoc* genus and in the present study, MIC was therefore read both at 24 and 48 hours (Clinical and Laboratory Standards Institute (CLSI), 2016). Overall, the *Leuconostoc* strains showed adequate growth at 24 hours, except for one *L. falkenbergense* strain and one *L. mesenteroides* strain, which showed limited growth at 24 hours; therefore, incubation for 48 hours was required for these two strains. For the remaining strains the MIC increased no more than two 2-fold dilutions between the 24 and 48 hours reading, and the population MIC range only increased one 2-fold dilution for most of the tested antimicrobial agents (chloramphenicol, clindamycin, erythromycin, gentamycin, kanamycin, streptomycin, tetracycline). Based on the results in the present study, MIC recording at 24 hours is recommended, since most of the strains showed adequate growth at this timepoint. However, in cases where poor growth is observed for specific strains it is recommended to incubate for 48 hours to obtain the correct MIC values.

**Table 4:** MIC distribution and tentative ECOFFs for nine antimicrobial agents for the *Leuconostoc* species.

	Species	Distribution (%) of MICs																		Tentative		
		0.0075	0.015	0.03	0.06	0.12	0.25	0.5	1	2	4	8	16	32	64	128	256	512	1024	ECOFF	MIC50	MIC90
Ampicillin	*L. falkebergense*(15)/*L. pseudomesenteroides*(2)					12	6	35	47											1	0.5	1
	*L. mesenteroides* (26)					15	19	23	27	15										2	0.5	2
Chloramphenicol	*L. falkebergense*(15)/*L. pseudomesenteroides*(2)									12	70	18								8	4	8
	*L. mesenteroides* (26)								15	46	39									4	2	4
Clindamycin	*L. falkebergense*(15)/*L. pseudomesenteroides*(2)		23		6	12			6		23	29								8	4	8
	*L. mesenteroides* (26)		73		27															0.06	≤0.03	0.06
Erythromycin	*L. falkebergense*(15)/*L. pseudomesenteroides*(2)			6	23	65	6													0.25	0.12	0.12
	*L. mesenteroides* (26)	** **		27	38	31	4													0.25	0.06	0.12
Gentamycin	*L. falkebergense*(15)/*L. pseudomesenteroides*(2)					18		41	23	18										2	0.5	2
	*L. mesenteroides* (26)					69		23	8											1	≤0.25	0.5
Kanamycin	*L. falkebergense*(15)/*L. pseudomesenteroides*(2)							12			6	35	23	23						32	8	32
	*L. mesenteroides* (26)							39		23	12	8	15	4						32	2	16
Streptomycin	*L. falkebergense*(15)/*L. pseudomesenteroides*(2)							6			18	41	18	18						32	8	32
	*L. mesenteroides* (26)							30		31		12	23	4						32	2	16
Tetracycline	*L. falkebergense*(15)/*L. pseudomesenteroides*(2)				6		6	18	47	18	6									4	1	2
	*L. mesenteroides* (26)						8	34	35	19	4									4	1	2
Vancomycin	*L. falkebergense*(15)/*L. pseudomesenteroides*(2)													100							>16	>16
	*L. mesenteroides* (26)													100							>16	>16

MIC is compared to the Leuconostoc microbial cut-off values by EFSA (vertical line). The white area shows the testedconcentration of the specific antimicrobials and the grey area shows the concentration of the specific antimicrobials not tested.

In the present study, the chloramphenicol, clindamycin and kanamycin MIC range was higher than the current cut-offs provided by EFSA (**Table 4**), which could be due to the difference in test conditions in the present study and previous published data (Swenson et al., 1990; Casado Muñoz M del et al., 2014; Jeong and Lee, 2015; Flórez et al., 2016).

Overall, the MIC distributions for *L. falkebergense*/*L. pseudomesenteroides* and *L. mesenteroides* were similar, expect for clindamycin.

The clindamycin MIC distribution for the *L. falkebergense*/*L. pseudomesenteroides* group was found to be divided into two subgroups with either clindamycin MIC at or below the current cut-off value of 1 mg/L (≤0.03-1 mg/L) and strains with MICs above (4-8 mg/L), respectively. The type strains of both species showed clindamycin MIC values of 8 mg/L, suggesting that decreased clindamycin susceptibility is an inherent trait of both species originating before species differentiation. In agreement, strains with clindamycin MIC above the current cut-off value were scattered throughout the phylogenetic tree (**Figure 2**) but the trait appears to have been lost from specific strains. Genome comparisons of clindamycin resistant and susceptible strains did not identify any evidence of acquired genes that could explain the resistance, supporting that decreased clindamycin susceptibility is intrinsic for the *L. falkebergense*/*L. pseudomesenteroides* group. A gene encoding a protein with 51.8% similarity to LsaA of *E. faecalis* has been suggested to be involved in the clindamycin resistance observed for the *L. pseudomesenteroides* type strain (Salvetti et al., 2021). However, this gene was found in all 17 strains included in the present study including strains with clindamycin MIC values below the EFSA cut-off value. Furthermore, whereas the intact 1,448 bp gene was present in some strains with low clindamycin MIC values, all *L. falkenbergense* strains with clindamycin MIC values above the EFSA cut-off value were found to encode a truncated 333 bp pseudogene due to a premature stop codon. Accordingly, the *lsaA*-like gene cannot explain the decreased clindamycin susceptibility. As there are no indications that the decreased clindamycin susceptibility commonly observed in strains of the *L. falkebergense*/*L. pseudomesenteroides* group is related to acquired genes, this can be considered as an inherent trait of the species and the clindamycin ECOFF should be adjusted to reflect this (**Table 4**).

### Detection of known antibiotic resistance genes

For all strains included in the study, the presence of genes with identity to known antimicrobial resistance genes was assessed using the curated databases ResFinder (Zankari et al., 2012) (nucleotide level) and NCBI AMRFinderPlus (Feldgarden et al., 2019) (amino acid level).

Out of the 170 included strains, correlation between phenotypic and genotypic resistance was only observed for one *L. sakei* strain (Accession number JANRGY000000000), which exhibit highly elevated tetracycline MIC compared to the wild-type population (**Table 1**) and encodes acquired tetracycline resistance genes (**Figure S1**) as described above.

In the remaining strains, no antibiotic resistance genes were detected using the EFSA cut-offs (% identity and coverage above 80% and 70%, respectively) (European Food Safety Authority, 2021). This supports that the decreased antimicrobial susceptibility observed in some of the species is an innate tolerance to specific antimicrobial agents. Innate tolerance or intrinsic resistance does not normally spread horizontally between bacteria but spreads clonally and is often seen as a common trait within a bacterial species or subpopulation which share a common evolutionary history (Cox and Wright, 2013).

## Conclusions

ECOFFs are a useful tool to differentiate susceptible and resistant strains within species, however MIC data on species level determined using a standardized method need to be available. In the present study, we were able to show that antimicrobial susceptibility for the *Lactobacilliaceae* family follows phylogeny and tentative ECOFFs were defined accordingly. Furthermore, the data shows that several of the current cut-offs defined by EFSA are either too high or too low for specific species and that several of the species exhibit intrinsic resistance towards specific antimicrobial agents, e.g., *L. pseudomesenteroides/falkenbergense* toward clindamycin and *L. salivarius* toward kanamycin. Furthermore, correlation between phenotypic resistance and presence of known antibiotic resistance genes was observed for one *L. sakei* strain out of the 170 included strains. Therefore, it is important that future tentative ECOFFs are defined based on phylogeny and that more data become available to define ECOFFs. When defining tentative ECOFFs for industrially relevant bacterial species the number of isolates available are often limited compared to clinically important species. It is therefore important; 1) that strains are unambiguously defined at species level and subtyped to support a diverse strain collection e.g., through core genome analysis, 2) MIC population studies are performed by use of a standardized method to define species-specific MIC distributions and 3) that the presence of known antimicrobial resistance genes are searched for to support the MIC distributions.

## Data availability statement

The datasets presented in this study can be found in online repositories. The names of the repository/repositories and accession number(s) can be found below: https://www.ncbi.nlm.nih.gov/genbank/, SAMN33225755-SAMN33225768, https://www.ncbi.nlm.nih.gov/genbank/, JANRGY000000000.

## Author contributions

KN-M produced the data, wrote the manuscript, made figures, tables, performed the analysis, and was involved in developing the concept and the method. CS was involved in developing the concept, guiding the analysis, discussion, review and editing. HI was involved in developing the concept, discussion, review, and editing. YA was involved in conceiving the idea, developing, and guiding the concept, analysis, design, discussion, review and editing. AK and KA-N did the sequencing and generation of genome assemblies. All authors contributed to the article and approved the submitted version.
