# Supplementary material for: Antimicrobial susceptibility testing and tentative epidemiological cut-off values for Lactobacillaceae family species intended for ingestion
Source: Front Antibiot. 2023 Jun 15;2:1162636. doi: 10.3389/frabi.2023.1162636 (PMC11732008; doi:10.3389/frabi.2023.1162636)

***Supplementary Material***

**Antimicrobial susceptibility testing and tentative epidemiological cut-off values for Lactobacillaceae family species intended for ingestion**

**Katrine Nøhr-Meldgaard, Carsten Struve, Hanne Ingmer, Anna Koza, Kosai Al-Nakeeb & Yvonne Agersø***

*** Correspondence:** Yvonne Agersø: DKYVAG@chr-hansen.com

Table S1: The origin, source, and year of isolation of the strains included in the study

| **Species** | **Type strain** | **Source** | **Year of isolation** | **Geographic area** |
| --- | --- | --- | --- | --- |
| ***Leuconostoc*** |  |  |  |  |
| *Leuconostoc mesenteroides* (26) | subsp. *mesenteroides* DSM20343  subsp. *cremoris* DSM20346 | Fermented milk product (7)  Camel’s milk (1)  Vegetable juice (1)  Fermented vegetable (2)  Fermenting olives (1)  Dried starter powder (1)  Not given (13) | 1961 (3), 1983 (3), ≤2012 (1)  ≤2012  ≤2014  ≤1986 (2)  ≤2008  ≤2000  1985 (1), ≤2005 (1), ≤2016 (1), ≤2017 (10) | Finland (3), Denmark (3), Mongolia (1)  Mongolia  Sweden  Not given (2)  Not given  Not given  Not given (13) |
| *Leuconostoc pseudomesenteroides* (2) | DSM20193 | Cane juice (1)  Forage crop (1) | ≤2000  1998 | Not given  Not given |
| *Leuconostoc falkenbergense* (15) | LMG10779 | Fermented milk product (4)  Kefir (3)  Not given (8) | 1961 (1), 1983 (1), 1990 (1), ≤2007 (1)  ≤2014 (3)  ≤1993 (1), ≤1997 (1), ≤1999 (1), ≤2004 (1), ≤2005 (1), ≤2006 (1), ≤2007 (1), ≤2012 (1) | Sweden (2), Not given (2)  Not given (3)  Not given (8) |
| ***Pediococcus*** |  |  |  |  |
| *Pediococcus acidilactici* (21) | NCFB2767 | Fermented milk product (3)  Silage (3)  Fermented meat (7)  Not given (8) | 2008 (1), ≤2013 (2)Ehrmann almighurtEhrmann almighurt  ≤1985 (2), ≤2003 (1)  ≤1987 (1), ≤1990 (1), 2001 (4), ≤2003 (1)  ≤1940 (1), ≤1987 (1), ≤1994 (1), ≤1999 (1), ≤2003 (2), ≤2010 (1), ≤2014 (1), | Not given (3)  Ireland (2), Not given (1)  Canada (1), Ireland (1), USA (2), Not given (3)  Ireland (2), Not given (6) |
| *Pediococcus pentosaceus* (21) | LMG114-88 | Fermented milk product (3)  Sake (1)  Dried beer yeast (1)  Fermented meat (4)  Silage (3)  Bakery culture (5)  Not given (4) | ≤2003 (1), ≤2012 (2)  ≤2007  ≤2003  ≤1983 (1), ≤1994 (1), 2001 (2)  ≤1984 (1), 1994 (1), ≤2003 (1)  ≤1983 (1), 1988 (1), ≤1988 (1), ≤1992 (1), ≤1994 (1)  ≤1983 (1), ≤1986 (1), ≤2011 (1), ≤2015 (1) | Ireland (1), Not given (2)  Not given  Ireland  Germany (1), USA (2), Not given (1)  Not given (3)  Germany (2), Italy (1), France (1), not given (1)  Spain (1), Not given (3) |
| ***Lactobacillus*** |  |  |  |  |
| *Lactobacillus* *gasseri* (7) | DSM20243 | Human feces (4)  Human blood, clinical isolate (2)  Human (1) | ≤2004 (1), ≤2012 (3)  2007 (1), 2010 (1)  ≤1996 | Denmark (1), Not given (3)  Denmark (2)  Not given |
| *Lactobacillus paragasseri* (9) | LMG11478 | Human blood, clinical isolate (1)  Human feces (5)  Yoghurt (1)  Not given (2) | 2004  1934 (1), ≤2005 (1), ≤2012 (3)  ≤2001  ≤2010 (1), ≤2017 (1) | Denmark  Japan (1), Denmark (1), Not given (3)  Japan  Not given (2) |
| *Lactobacillus helveticus* (7) | DSM20075 | Fermented milk product (4)  Starter culture (1)  Not given (2) | 1986 (1), 1991 (1), 2011 (1), ≤2013 (1)  1983  ≤1993 (1), Not given (1) | Albania, Argentina (2), Not given  France  Not given (2) |
| *Lactobacillus delbrueckii* subsp. *bulgaricus* (19) | subsp. *bulgaricus* DSM20081 | Yogurt (9)  Milk (1)  Not given (9) | 1985 (1), ≤ 1986 (2), 1986 (1), ≤1990 (1), ≤ 1996 (1), ≤2014 (2), ≤ 2016 (1)  ≤ 2007  1986 (1), ≤ 1992 (1), ≤ 1994 (1), ≤ 1995 (1), ≤ 1998 (1), ≤ 1999 (1), ≤ 2001 (1), ≤ 2006 (1), ≤ 2019 (1) | Albania (1), Bulgaria (4), Netherlands (1), not given (3)  Israel  Not given (9) |
| *Lactobacillus delbrueckii* subsp. *lactis* (9) | subsp. *lactis* DSM20072 | Raw milk cheese (1)  Cheese (1)  Not given (7) | ≤ 2002  ≤ 1990  ≤ 1998 (2), ≤ 1993 (1), ≤ 2000 (1), ≤ 2005 (1), ≤ 2010 (1) ≤ 2011 (1) | Not given  Not given  France (1), Italy (1), Not given (5) |
| ***Latilactobacillus*** |  |  |  |  |
| *Latilactobacillus sakei* (10) | LMG9468 | Raw milk (1)  Human feces (1)  Chicken meat (1)  Meat (4)  Starter of sake (1)  Not given (2) | 2004  ≤ 2012  ≤ 1993  ≤1987 (1), ≤ 1993 (2), 2010 (1)  ≤ 1999  ≤ 2012 (2) | Italy  Not given  Not given  Denmark (1), Not given (3), France (1)  Not given  Not given (2) |
| ***Lentilactobacillus*** |  |  |  |  |
| *Lentilactobacillus parabuchneri* (9) | LMG11457 | Brewery yeast (1)  Blair athol distillery (1)  Cheese (3)  Human saliva (2)  Not given (2) | ≤ 1989  ≤ 2002  ≤ 1956 (1), ≤ 2004 (1), ≤ 2018 (1)  ≤ 1960 (2)  ≤ 1993 (1), ≤ 2008 (1) | Not given  Not given  Denmark (1), Finland (1), not given (1)  United Kingdom (2)  Not given (2) |
| ***Ligilactobacillus*** |  |  |  |  |
| *Ligilactobacillus salivarius* (12) | LMG9477 | Chicken (1)  Animal feed (1)  Human feces (5)  Human blood, clinical isolate (1)  Not given (4) | ≤ 1986  ≤ 1995  ≤ 2005 (1), ≤ 2010 (2), ≤ 2012 (2)  2007  ≤ 2004 (1), ≤ 2006 (1), not given (2) | Not given  Not given  Not given (5)  Denmark  Not given (4) |
| ***Limosilactobacillus*** |  |  |  |  |
| *Limosilactobacillus fermentum* (13) | LMG6902 | Human blood, clinical isolate (5)  Human feces (1)  Rennet (1)  Probiotic (1)  Fermented milk product (1)  Fermented beets (1)  Corn oil (1)  Ascites (1)  Not given (1) | 2006 (2), 2007 (1), 2009 (1), 2010 (1)  ≤ 2012  1956  ≤ 2012  ≤ 2002  ≤ 2005  ≤ 1986  2005  ≤ 1985 | Denmark (5)  Not given  Not given  Not given  Not given  Not given  Not given  Denmark  Not given |

Table S2: Comparison of minimal inhibitory concentration measured with VetMIC and Sensititre panel

|  | Ampicillin | | Chloramphenicol | | Clindamycin | | Erythromycin | | Gentamycin | | Kanamycin | | Streptomycin | | Tetracycline | | Vancomycin | |
| --- | --- | --- | --- | --- | --- | --- | --- | --- | --- | --- | --- | --- | --- | --- | --- | --- | --- | --- |
|  | VetMIC | Sensititre | VetMIC | Sensititre | VetMIC | Sensititre | VetMIC | Sensititre | VetMIC | Sensititre | VetMIC | Sensititre | VetMIC | Sensititre | VetMIC | Sensititre | VetMIC | Sensititre |
| *Pediococcus acidilactici* (20 hours) |  |  |  |  |  |  |  |  |  |  |  |  |  |  |  |  |  |  |
|  | 4 | 4 | 8 | 4 | 0.12 | <0.03 | 0.5 | 0.25 | 4 | 4 | 128 | 128 | 64 | 64 | 32 | 16 | >128 | >16 |
|  | 4 | 4 | 8 | 4 | 0.06 | <0.03 | 0.25 | 0.25 | 4 | 4 | 128 | 128 | 64 | 64 | 32 | 16 | >128 | >16 |
|  | 4 | 2 | 8 | 4 | 0.12 | 0.06 | 0.5 | 0.25 | 4 | 8 | 64 | 128 | 64 | 64 | 16 | 32 | >128 | >16 |
|  | 1 | 1 | 8 | 4 | 0.06 | <0.03 | 0.25 | 0.06 | 2 | 2 | 64 | 32 | 32 | 32 | 16 | 16 | >128 | >16 |
|  | 2 | 2 | 8 | 4 | 0.06 | <0.03 | 0.25 | 0.25 | 4 | 4 | 128 | 64 | 64 | 64 | 16 | 16 | >128 | >16 |
| *Pediococcus pentosaceus* (20 hours) |  |  |  |  |  |  |  |  |  |  |  |  |  |  |  |  |  |  |
|  | 4 | 4 | 4 | 4 | 0.12 | 0.12 | 0.25 | 0.12 | 4 | 4 | 128 | 128 | 64 | 64 | 8 | 16 | 128 | >16 |
|  | 8 | 8 | 4 | 8 | 0.06 | <0.03 | 0.25 | 0.12 | 2 | 2 | 64 | 32 | 32 | 32 | 16 | 16 | 128 | >16 |
|  | 4 | 4 | 4 | 4 | 0.06 | <0.03 | 0.25 | 0.25 | 4 | 4 | 64 | 64 | 64 | 64 | 16 | 16 | 128 | >16 |
| *Leuconostoc mesenteroides* (24 hours) |  |  |  |  |  |  |  |  |  |  |  |  |  |  |  |  |  |  |
|  | 2 | 2 | 4 | 8 | 0.06 | 0.06 | 0.25 | 0.25 | NA | 0.5 | 16 | 16 | 8 | 16 | 2 | 4 | >128 | >16 |
|  | 1 | 1 | 4 | 4 | 0.06 | 0.06 | 0.12 | 0.12 | 0.5 | <0.25 | 8 | 8 | 4 | 8 | 1 | 1 | >128 | >16 |
|  | 1 | 1 | 8 | 4 | 0.06 | 0.06 | 0.12 | 0.12 | 1 | 1 | 16 | 16 | 16 | 32 | 2 | 2 | >128 | >16 |
| *Leuconostoc pseudomesenteroides* (24 hours) |  |  |  |  |  |  |  |  |  |  |  |  |  |  |  |  |  |  |
|  | 2 | 1 | 4 | 4 | 4 | 8 | 0.12 | 0.25 | 1 | 1 | 32 | 32 | 16 | 32 | 1 | 1 | >128 | >16 |
| *Leuconostoc falkenbergense* (24 hours) |  |  |  |  |  |  |  |  |  |  |  |  |  |  |  |  |  |  |
|  | 1 | 1 | 8 | 4 | 16 | 8 | 0.5 | 0.12 | 4 | 2 | 128 | 64 | 64 | 32 | 4 | 4 | >128 | >16 |
|  | 1 | 1 | 8 | 8 | 0.12 | 0.25 | 0.12 | 0.12 | NA | 1 | 16 | 16 | 8 | 16 | 4 | 4 | >128 | >16 |
|  | 0.25 | 0.5 | 4 | 4 | 4 | 4 | 0.25 | 0.12 | 1 | 0.5 | 16 | 16 | 8 | 8 | 1 | 0.5 | >128 | >16 |
|  | 0.5 | 0.5 | 2 | 4 | 1 | 1 | 0.06 | 0.06 | <0.3 | 0.5 | 4 | 8 | 4 | 4 | 0.5 | 0.25 | >128 | >16 |
| *Lactobacillus delbrueckii* subsp. *bulgaricus* |  |  |  |  |  |  |  |  |  |  |  |  |  |  |  |  |  |  |
|  | 0.06 | 0.06 | 4 | 4 | 0.06 | <0.03 | 0.03 | <0.015 | 4 | 4 | 128 | 64 | 16 | 16 | 1 | 1 | 0.5 | 0.25 |
|  | 0.12 | 0.06 | 8 | 4 | 0.12 | 0.06 | 0.06 | 0.06 | 2 | 2 | 32 | 16 | 8 | 4 | 4 | 2 | 0.25 | 0.25 |
|  | 0.06 | <0.03 | 4 | 2 | 0.12 | 0.06 | 0.06 | 0.03 | 1 | 1 | 8 | 8 | 8 | 4 | 4 | 1 | <0.25 | 0.25 |
|  | 0.06 | 0.06 | 2 | 2 | 0.12 | 0.06 | 0.06 | 0.03 | 0.5 | 0.5 | 2 | 2 | 1 | 2 | 1 | 1 | 0.25 | 0.5 |
|  | 0.06 | <0.03 | 4 | 4 | 0.12 | 0.06 | 0.03 | 0.03 | 1 | 1 | 8 | 4 | 4 | 4 | 2 | 1 | 0.25 | 0.25 |
|  | 0.12 | 0.12 | 2 | 4 | 0.12 | 0.12 | <0.016 | 0.03 | 2 | 2 | 32 | 32 | 4 | 8 | 1 | 1 | 0.5 | 0.5 |
| *Lactobacillus delbrueckii* subsp. *lactis* |  |  |  |  |  |  |  |  |  |  |  |  |  |  |  |  |  |  |
|  | 0.12 | 0.12 | 4 | 4 | 0.25 | 0.06 | 0.12 | 0.12 | 1 | 1 | 16 | 16 | 4 | 4 | 4 | 2 | 0.5 | 0.5 |
| *Ligilactobacillus salivarius* |  |  |  |  |  |  |  |  |  |  |  |  |  |  |  |  |  |  |
|  | 1 | 0.5 | 4 | 4 | 1 | 1 | 0.25 | 0.25 | 1 | 4 | 64 | 128 | 16 | 32 | 4 | 4 | >128 | >16 |
| *Lactilactobacillus sakei* |  |  |  |  |  |  |  |  |  |  |  |  |  |  |  |  |  |  |
|  | 2 | 4 | 4 | 4 | 1 | 1 | 0.25 | 0.25 | 8 | 8 | 32 | 32 | 64 | 128 | 4 | 4 | >128 | >16 |

NA: not available

Table S3: MIC distribution and tentative ECOFFs for nine antimicrobial agents for the obligate heterofermentative species

| Antimicrobial agent | Species | Distribution (%) of MICs | | | | | | | | | | | | | | | | | | Tentative |  |  |
| --- | --- | --- | --- | --- | --- | --- | --- | --- | --- | --- | --- | --- | --- | --- | --- | --- | --- | --- | --- | --- | --- | --- |
|  |  | 0.0075 | 0.015 | 0.03 | 0.06 | 0.12 | 0.25 | 0.5 | 1 | 2 | 4 | 8 | 16 | 32 | 64 | 128 | 256 | 512 | 1024 | ECOFF | MIC50 | MIC90 |
| Ampicillin | *Lentilactobacillus parabuchneri* (9) |  |  |  |  |  |  | 11 | 33 | 56 |  |  |  |  |  |  |  |  |  | 2 | 2 | 2 |
|  | *Limosilactobacillus fermentum* (13) |  |  |  |  | 54 | 8 | 30 | 8 |  |  |  |  |  |  |  |  |  |  | 1 | 0.12 | 0.5 |
| Chloramphenicol | *Lentilactobacillus parabuchneri* (9) |  |  |  |  |  |  |  |  |  | 22 | 78 |  |  |  |  |  |  |  | 8 | 8 | 8 |
|  | *Limosilactobacillus fermentum* (13) |  |  |  |  |  |  |  |  |  |  | 92 | 8 |  |  |  |  |  |  | 16 | 8 | 8 |
| Clindamycin | *Lentilactobacillus parabuchneri* (9) |  | 100 |  |  |  |  |  |  |  |  |  |  |  |  |  |  |  |  | ≤0.03 | ≤0.03 | ≤0.03 |
|  | *Limosilactobacillus fermentum* (13) |  | 100 |  |  |  |  |  |  |  |  |  |  |  |  |  |  |  |  | ≤0.03 | ≤0.03 | ≤0.03 |
| Erythromycin | *Lentilactobacillus parabuchneri* (9) |  |  |  |  | 44 | 44 | 11 |  |  |  |  |  |  |  |  |  |  |  | 0.5 | 0.25 | 0.25 |
|  | *Limosilactobacillus fermentum* (13) |  |  |  |  |  | 62 | 38 |  |  |  |  |  |  |  |  |  |  |  | 0.5 | 0.25 | 0.5 |
| Gentamycin | *Lentilactobacillus parabuchneri* (9) |  |  |  |  | 67 |  | 22 | 11 |  |  |  |  |  |  |  |  |  |  | 1 | ≤0.12 | 1 |
|  | *Limosilactobacillus fermentum* (13) |  |  |  |  |  |  |  | 54 | 31 | 15 |  |  |  |  |  |  |  |  | 4 | 1 | 4 |
| Kanamycin | *Lentilactobacillus parabuchneri* (9) |  |  |  |  |  |  |  |  |  | 22 | 44 | 22 | 11 |  |  |  |  |  | 32 | 8 | 32 |
|  | *Limosilactobacillus fermentum* (13) |  |  |  |  |  |  |  |  |  |  |  |  | 69 | 31 |  |  |  |  | 64 | 32 | 64 |
| Streptomycin | *Lentilactobacillus parabuchneri* (9) |  |  |  |  |  |  |  |  |  | 44 | 44 | 11 |  |  |  |  |  |  | 16 | 8 | 16 |
|  | *Limosilactobacillus fermentum* (13) |  |  |  |  |  |  |  |  |  |  |  | 38 | 54 | 8 |  |  |  |  | 64 | 32 | 32 |
| Tetracycline | *Lentilactobacillus parabuchneri* (9) |  |  |  |  |  |  |  |  |  |  |  | 67 | 22 | 11 |  |  |  |  | 64 | 16 | 64 |
|  | *Limosilactobacillus fermentum* (13) |  |  |  |  |  |  |  |  |  |  | 63 | 38 |  |  |  |  |  |  | 16 | 8 | 16 |
| Vancomycin | *Lentilactobacillus parabuchneri* (9) |  |  |  |  |  |  |  |  |  |  |  |  | 100 |  |  |  |  |  |  | >16 | >16 |
|  | *Limosilactobacillus fermentum* (13) |  |  |  |  |  |  |  |  |  |  |  |  | 100 |  |  |  |  |  |  | >16 | >16 |

Figure S1: Genomic position of tetracycline resistance genes in tetracycline resistant *L. sakei* strain


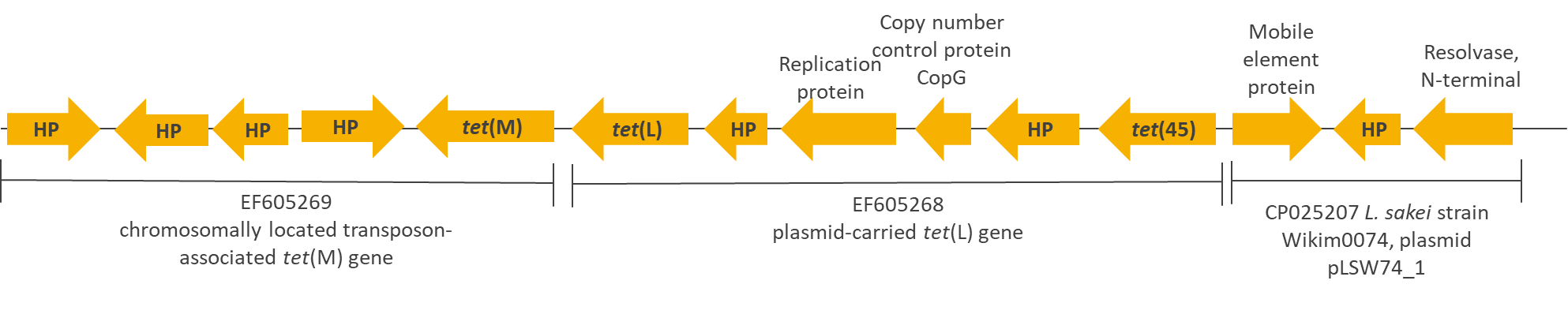

Supplement: Supplementary file 1 [file DataSheet_1.docx]
